# Discordance in the 2018 Periodontal Classification: Conceptual Challenges and a Biologically Grounded Framework for Interpretation

**DOI:** 10.3390/dj14060374

**Published:** 2026-06-16

**Authors:** Nada Tawfig Hashim, Bakri Gobara Gismalla, Bhavna Jha Kukreja, Ayman Ahmed, Nallan C. S. K. Chaitanya, Salma Musa Adam Abduljalil, Hiba Ahmed Elsidig, Muhammed Mustahsen Rahman

**Affiliations:** 1 Department of Periodontics, RAK College of Dental Sciences, RAK Medical & Health Sciences University, Ras-AL Khaimah 12973, United Arab Emirates; 2 Department of Oral Rehabilitation, Faculty of Dentistry, University of Khartoum, Khartoum 11115, Sudan; 3 Department of Preventive Dental Sciences, College of Dentistry, Gulf Medical University, Ajman P.O. Box 4184, United Arab Emirates; 4 Department of Periodontology and Implantology, College of Dentistry, The National Ribat University, Khartoum 11115, Sudan; 5 Department of Oral Medicine and Radiology, RAK College of Dental Sciences, RAK Medical & Health Sciences University, Ras-AL Khaimah 12973, United Arab Emirates; 6 Department of Periodontology, Faculty of Dentistry, National University, Khartoum 11115, Sudan; 7 Department of Periodontology and Implantology, Nile University, Khartoum 11115, Sudan

**Keywords:** periodontitis classification, staging, grading, localized and generalized periodontitis, smoking, diabetes, bone loss, clinical phenotypes, periodontal diagnosis, conceptual framework

## Abstract

The 2018 classification of periodontal and peri-implant diseases introduced a multidimensional diagnostic framework integrating staging, grading, and disease extent, representing a major advance over earlier severity-based systems. By incorporating structural destruction, treatment complexity, spatial distribution, and estimated risk of progression, the classification aimed to support more individualized and biologically informed diagnosis. However, increasing clinical application has revealed interpretive challenges, particularly in cases where different components of the system appear discordant. This perspective examines these challenges through a conceptual and clinical lens, focusing on the distinction between focal severity and overall disease burden in staging, the biological meaning of disease distribution, the interpretation of tooth loss as a historical rather than current indicator of disease status, and the need to differentiate between observed progression and risk-based modifiers in grading. Rather than reflecting deficiencies of the classification itself, these discordances are understood as a consequence of applying categorical systems to a biologically heterogeneous and temporally dynamic disease. A biologically grounded interpretive hierarchy is proposed, prioritizing observed tissue behavior and realized tissue destruction over probabilistic risk indicators while integrating structural parameters, historical outcomes, and susceptibility modifiers within their appropriate conceptual roles. This approach enhances diagnostic coherence and supports a more phenotype-oriented interpretation of periodontal disease.

## 1. Introduction

The 2018 classification of periodontal and peri-implant diseases (hereafter “the 2018 classification”, derived from the 2017 World Workshop, whose consensus reports were published in 2018) represents a major advancement in periodontal diagnosis, introducing a multidimensional framework that integrates staging, grading, and disease extent [1]. By distinguishing between the severity and complexity of tissue destruction, the rate and risk of disease progression, and the spatial distribution of affected sites, the system reflects a more comprehensive understanding of periodontitis as a biologically heterogeneous condition [2].

A central conceptual strength of this framework lies in its transition from purely descriptive, severity-based categorization toward a multidimensional representation of disease expression. By separating stage from grade, the classification acknowledges that the magnitude of existing tissue destruction and the biological behavior of disease progression are not equivalent constructs. Similar levels of attachment loss may arise through distinct temporal patterns and under varying host and environmental influences [3]. Accordingly, the framework extends beyond retrospective assessment of structural damage and incorporates considerations of treatment complexity and anticipated disease trajectory. However, this conceptual advancement also introduces interpretive challenges, as the system must accommodate a disease that is spatially uneven, temporally non-linear, and influenced by both local ecological factors and systemic conditions [3,4].

With increasing clinical application, situations have emerged in which different components of the classification appear discordant. These scenarios typically arise when current tissue destruction, historical outcomes such as tooth loss, and modifier-based grading do not align, creating ambiguity in diagnostic interpretation. Importantly, such discordance does not reflect inherent deficiencies of the classification itself but rather the difficulty of translating a continuous and multifactorial biological process into discrete diagnostic categories. Periodontitis does not follow a uniform or deterministic course; instead, it manifests as a spectrum of phenotypes shaped by dynamic interactions between the biofilm, host immune response, and systemic influences [1,2].

In a clinical population in a sub-Saharan setting, application of the 2018 classification yielded a high proportion of advanced-stage cases, illustrating challenges in translating the criteria into diverse clinical settings [5]. Such distributions should be interpreted with caution, however, because the apparent severity profile is sensitive to the population sampled and to the case definition and recording protocol used: representative epidemiological data indicate that the 2018 framework can overestimate periodontitis prevalence relative to established case definitions, while partial-mouth recording can distort the apparent distribution of disease severity [6].

Recent discussions have advocated for expanding grade modifiers to include additional systemic and inflammatory parameters. While this approach may enhance the granularity of risk assessment, it also introduces greater complexity [7,8]. The accumulation of multiple modifiers may generate risk profiles that do not consistently correspond to the patient’s observed clinical presentation. This raises an important question: whether increasing the number of modifiers necessarily improves diagnostic clarity or, conversely, complicates clinical interpretation [7]. This challenge becomes particularly relevant when the classification is used not only as a descriptive system but also as a tool for prognosis estimation and treatment planning. In such contexts, discrepancies between structural destruction, disease distribution, historical burden, and risk modifiers require careful interpretation, ideally within a hierarchical framework rather than as equivalent indicators [9,10].

Accordingly, the present perspective does not aim to expand existing classification criteria but rather to re-examine how these components should be interpreted within a biologically anchored approach. In this perspective, “biologically grounded” is used in a specific and limited sense: it denotes anchoring diagnostic interpretation in observed tissue behavior—the realized destruction actually present in the periodontium—rather than in probabilistic risk. It is therefore a phenotype-level claim rather than a molecular, immunologic, or microbiologic one; the integration of molecular, immunologic, microbiologic, and biomarker evidence is acknowledged as a future direction beyond the present phenotype-oriented framework. The discussion focuses on key areas in which discordance may arise and proposes a structured interpretive hierarchy that prioritizes observed tissue behavior while contextualizing structural parameters, historical outcomes, and susceptibility modifiers. Although the present analysis focuses primarily on periodontitis, the same interpretive tension is raised here only as an analogy to peri-implant conditions, where a structural outcome and current disease activity may not always align; systematic application of the proposed hierarchy to peri-implant diagnosis is left to dedicated future work. These considerations further extend to the definition of gingival health within the same classification system, where threshold-based criteria may not fully capture site-specific inflammatory changes. This highlights a broader limitation of categorical diagnostic models: while essential for standardization and communication, they may not adequately represent the inherent variability in and dynamic nature of biological disease processes.

Previous work has focused on clarifying the clinical application of the classification and identifying practical “gray zones” in its implementation. The present perspective extends beyond application to examine the underlying conceptual basis of these discrepancies and proposes a biologically anchored interpretive approach [8].

This paper adopts a structured narrative approach to examine conceptual and clinical challenges associated with the application of the 2018 periodontal classification. Relevant literature was identified through targeted searches of PubMed and Scopus covering the period January 2017 to January 2026, using the principal search terms “periodontitis classification”, “staging”, “grading”, “extent”, “grade modifiers”, and “diagnostic discordance/agreement”, alone and in combination. Searches were restricted to English-language publications. Sources were prioritized on the basis of relevance rather than exhaustiveness: the 2018 consensus reports and subsequent implementation and inter-examiner agreement studies were given precedence, while mechanistic sources were cited only to support specific statements. This is, by design, a non-systematic narrative selection and is not intended as an exhaustive or reproducible synthesis. The present work is a conceptual Perspective: it reports no original data, no quantitative or statistical analysis, and no systematic review, and its proposals are therefore interpretive. They are offered as hypotheses requiring empirical validation—for example, studies testing whether the proposed hierarchy improves inter-examiner agreement, prognostic accuracy, or treatment decisions—rather than as established findings.

## 2. Interpretive Framework

The interpretive framework proposed here is based on the premise that the components of periodontal classification do not all carry the same biological meaning. Some parameters primarily reflect realized tissue destruction, others describe the anatomical and spatial expression of disease, and others indicate susceptibility or historical burden. When these dimensions are interpreted as equivalent, diagnostic discordance becomes more likely. Conversely, when they are understood as biologically distinct yet complementary layers of information, apparently conflicting classifications can be reconciled in a more coherent and clinically meaningful manner [1,2]. This three-way distinction—between realized tissue destruction, historical burden, and risk modifiers—is established once here and is applied, rather than re-derived, in the sections that follow: each subsequent section advances a distinct point (Section 6, tooth loss as a historical endpoint; Section 7, progression versus risk in grading; Section 8, smoking and attenuated clinical signs; Section 9, resolving tissue-versus-modifier conflicts).

As Table 1 makes explicit, the present proposal adds an interpretive layer on top of the existing 2018 criteria rather than introducing new criteria: the formal definitions of stage, grade, extent, and modifiers are retained unchanged, and only their relative weighting when components conflict is specified.

## 3. Gingival Health and Threshold Limitations

The 2018 classification defines gingival health and gingivitis primarily based on the percentage of sites exhibiting bleeding on probing, with a threshold of less than 10% considered indicative of clinical gingival health. While this approach provides a standardized and reproducible method for patient-level classification, it may present interpretive challenges in certain clinical scenarios [11].

Periodontal inflammation is inherently site-specific, reflecting localized host–microbial interactions and variable inflammatory responses across different areas of the dentition. As a result, patients may exhibit clear clinical signs of gingival inflammation at individual sites while maintaining an overall bleeding score below the diagnostic threshold. In such cases, a patient may be classified as having gingival health at the global level despite the presence of localized inflammatory changes. This interpretation is anchored in the 2018 consensus definitions of periodontal health and plaque-induced gingivitis, which establish bleeding on probing as a site-level measure and adopt the patient-level threshold of fewer than 10% of bleeding sites as the criterion for clinical gingival health [11]. Applying a single patient-level threshold to a fundamentally site-level process is the source of the interpretive tension discussed here, rather than a deficiency of the consensus definition itself.

This issue is further accentuated in individuals with altered host responses, particularly smokers, in whom vascular and inflammatory changes reduce the expression of bleeding despite underlying inflammation. Consequently, reliance on a single quantitative threshold may not fully capture the biological variability in gingival inflammatory expression [12].

Threshold-based definitions remain essential for standardization in clinical and research settings; however, they should be interpreted as operational tools rather than comprehensive representations of the underlying biology. Localized inflammatory changes may still be clinically significant even when patient-level thresholds are not exceeded. This highlights a broader limitation of categorical systems in periodontal diagnosis, where continuous and site-specific processes are simplified into discrete classifications [13,14]. Accordingly, gingival health should be interpreted within a spectrum framework in which patient-level categorization provides a general guide, while site-level assessment remains essential for accurate clinical evaluation.

## 4. Staging: Focal Severity Versus Overall Disease Burden

Staging in the 2018 classification is determined by the most severely affected site, ensuring that advanced lesions are not underestimated [2]. This approach preserves diagnostic sensitivity for identifying complex cases requiring advanced management. However, the biological meaning of stage must be interpreted carefully, as it reflects the maximum severity of structural breakdown rather than the overall burden of disease across the dentition [1,2,3].

A key implication of this approach is that stage represents the peak expression of destruction, not its distribution. A single site exhibiting deep attachment loss, vertical bone defects, or furcation involvement may elevate the stage, even when the remaining dentition demonstrates relatively limited involvement [2]. In this sense, staging captures the highest level of anatomical and therapeutic complexity, rather than providing a summary measure of inflammatory load or disease extent. This distinction is critical in clinical interpretation [3]. Without it, there is a risk of equating localized structural severity with generalized disease burden, which may lead to overestimation of overall disease impact. Accordingly, stage should be understood as an indicator of maximum lesion severity and complexity, requiring contextual interpretation alongside other dimensions of the classification, particularly disease distribution.

## 5. Disease Distribution: Interpreting Extent Alongside Severity

Extent reflects the proportion of affected teeth and describes how periodontal destruction is distributed across the dentition. Unlike staging, which captures the severity of the most affected site, extent provides insight into the spatial organization of disease, distinguishing between localized and generalized patterns [1,2].

This distinction is biologically meaningful. Localized disease patterns typically indicate that destructive processes are confined to specific regions, often influenced by local ecological or anatomical factors. In contrast, generalized involvement reflects a broader disruption of host–microbial homeostasis, in which inflammatory and dysbiotic processes are expressed across multiple sites. These patterns represent different modes of disease expression, rather than variations in severity alone [15]. Importantly, extent does not modify stage but complements it by defining the distributional phenotype of the disease. A patient with localized severe destruction differs fundamentally from one with generalized moderate involvement, even if staging alone might suggest comparable severity. Therefore, extent should be interpreted as an independent dimension that characterizes how disease is organized across the dentition, rather than as a secondary descriptor of severity.

In addition to classification-related factors, diagnostic approaches used in routine clinical practice may also contribute to discrepancies between observed disease and assigned classification. The Basic Periodontal Examination (BPE), widely used as a screening tool, provides a simplified assessment based on sextant-level scoring rather than detailed site-specific measurements. While efficient for initial evaluation, this approach may overlook localized severe lesions or underestimate the extent of structural destruction, particularly in cases where advanced breakdown is confined to limited sites. Consequently, reliance on screening-based assessments without comprehensive periodontal charting may contribute to underrecognition of disease severity within the framework of the 2018 classification, further emphasizing the importance of detailed clinical evaluation in achieving accurate diagnostic interpretation [16,17].

## 6. Tooth Loss: Historical Outcome Versus Current Disease Status

**Figure 1:**
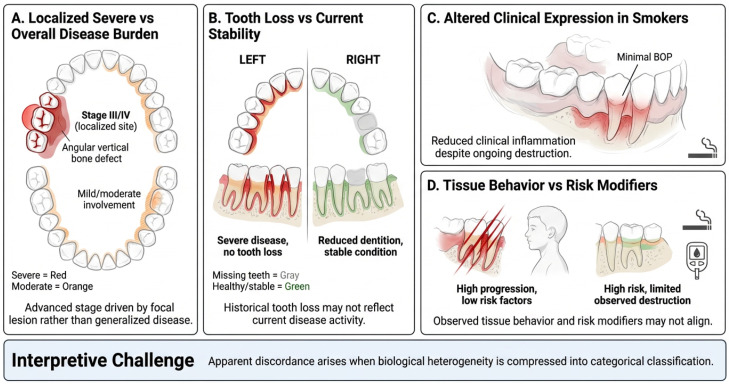
Clinical scenarios illustrating discordance in periodontal classification. Each panel pairs a discordance scenario with its clinical and radiographic or periodontal-charting correlate: (**A**) localized severe destruction determining stage assignment despite limited overall disease burden; (**B**) a reduced but stable dentition retaining a high stage on the basis of historical tooth loss; (**C**) attenuated bleeding on probing in a smoker despite ongoing destruction; and (**D**) a mismatch between observed tissue behavior and risk modifiers. Figure created with FigureLab.

Tooth loss is incorporated into staging as an indicator of cumulative disease impact; however, it fundamentally represents a historical outcome rather than a direct measure of current disease activity [2]. Its occurrence is influenced not only by the severity of periodontal destruction but also by clinical decision-making, timing of intervention, patient preferences, and access to care. As a result, tooth loss reflects the trajectory of past disease and treatment, and may not reliably correspond to the present biological state of the periodontium [18] (Figure 1).

This distinction becomes particularly important in discordant clinical scenarios, but it must be framed accurately with respect to the staging rules. In the 2018 classification, Stage III can be assigned on the basis of complexity factors alone—for example a probing depth of 6 mm or more, vertical bone loss of 3 mm or more, Class II or III furcation involvement, or a moderate ridge defect—independently of whether any teeth have been lost. Tooth loss due to periodontitis is itself one of several complexity criteria: the loss of four or fewer teeth is compatible with Stage III, whereas the loss of five or more teeth distinguishes Stage IV. Tooth loss therefore functions as an additional criterion that can escalate the stage—most relevant at the transition from Stage III to Stage IV—rather than as a prerequisite without which advanced current destruction goes unrecognized. A patient with extensive attachment loss, deep periodontal pockets, and vertical bone defects already qualifies for Stage III regardless of whether extractions have occurred. The discordance worth highlighting is therefore not a failure of staging to recognize advanced current destruction but the mismatch between past tooth loss and current disease activity: a stable, treated, reduced dentition may retain a high stage on the basis of historical extractions even when present inflammation is controlled [19].

Conversely, patients who have undergone extraction of hopeless teeth due to periodontitis may present with a reduced but relatively stable dentition, characterized by minimal residual pocketing and controlled inflammation. Despite a more favorable current clinical condition, such patients may be assigned a higher stage based on past tooth loss. These contrasting scenarios illustrate that tooth loss does not consistently align with present disease behavior, as it captures the consequences of prior disease activity rather than its current expression [20,21].

From a biological perspective, active periodontal destruction reflects ongoing host–microbial interactions, whereas tooth loss represents a downstream consequence of these processes [22]. Once extraction has occurred, the remaining dentition no longer necessarily reflects the inflammatory burden that led to tooth removal [23]. Similarly, preservation of teeth does not imply disease control, as advanced structural damage may persist in the absence of extraction. For this reason, tooth loss should be interpreted as a marker of disease legacy and cumulative impact, rather than as a direct surrogate for current severity [24].

Accordingly, an overreliance on tooth loss as a staging determinant may lead to misrepresentation of present disease status. Greater diagnostic emphasis should be placed on current clinical parameters, including attachment loss patterns, probing depth, and defect morphology, while tooth loss should be considered a contextual indicator of historical burden and functional complexity. Interpreting these elements within their appropriate biological roles allows for a more accurate alignment between classification and the dynamic reality of periodontal disease, particularly in cases where past outcomes and current tissue conditions diverge.

## 7. Grading: Distinguishing Progression from Risk

Grading within the 2018 classification is intended to estimate the rate of periodontal disease progression, reflecting the dynamic interaction between microbial challenge and host response. The most biologically relevant indicators of progression are derived from either direct longitudinal evidence of attachment or bone loss, or indirect estimation based on the relationship between bone loss and patient age [2,25]. These measures aim to approximate the velocity of tissue destruction, providing insight into how rapidly periodontal breakdown has occurred or is likely to occur [2].

However, the interpretation of grading is complicated by the temporal nature of periodontal disease. Rather than progressing in a continuous linear manner, periodontitis is increasingly understood as an episodic condition characterized by periods of relative stability interspersed with phases of active breakdown. As a result, similar bone loss–age ratios may reflect fundamentally different disease trajectories. One patient may exhibit early rapid destruction followed by long-term stability, while another may demonstrate recent acceleration of disease, despite comparable cumulative bone loss. This limitation highlights that indirect measures of progression do not fully capture the temporal dynamics of periodontal breakdown [25,26].

A critical conceptual distinction must therefore be maintained between observed progression and predicted risk. Indicators such as attachment loss patterns and bone loss relative to age reflect realized tissue outcomes, whereas factors such as smoking and glycemic status represent susceptibility modifiers that influence the probability of future progression. Although these modifiers are biologically significant, they do not constitute direct evidence that accelerated destruction has occurred in a given patient [27,28].

Failure to distinguish between these dimensions may lead to interpretive inconsistency, whereby grading is influenced disproportionately by exposure status rather than by actual patterns of tissue breakdown. For example, a patient with limited structural destruction but significant systemic risk factors may be assigned a higher grade, while another with pronounced age-disproportionate bone loss but fewer recognized modifiers may appear comparatively lower in grade. Such scenarios reflect not inconsistency in the classification itself but a conceptual conflation of risk potential with expressed disease behavior.

To address this, grading should be interpreted within a hierarchical framework in which evidence of tissue destruction serves as the primary anchor, and risk modifiers are used to contextualize, rather than redefine, the biological meaning of that destruction. This approach preserves the intended role of grading as an indicator of disease behavior while allowing for meaningful integration of systemic and behavioral influences. By maintaining this distinction, grading remains aligned with observed disease behavior and avoids over-reliance on probabilistic factors that may not correspond to current disease expression.

## 8. Smoking and Altered Clinical Expression

Smoking significantly modifies periodontal pathophysiology through its effects on vascular function, immune response, and oxidative stress. These changes reduce classical clinical signs of inflammation, such as bleeding on probing and gingival erythema, despite ongoing tissue destruction. As a result, clinical presentation may underestimate disease activity if assessment relies primarily on inflammatory indicators, while structural parameters—including attachment loss, probing depth, and radiographic bone levels—provide more reliable indicators of disease severity in such cases [29].

The biological basis of this altered presentation is critical to understanding its impact on classification. Smoking induces vasoconstriction, reduces gingival blood flow, and alters microvascular permeability, thereby attenuating the vascular response that underlies clinical bleeding. In parallel, it modulates neutrophil function, cytokine expression, and oxidative balance, resulting in a modified inflammatory profile that is less clinically visible despite ongoing tissue breakdown. These effects effectively uncouple overt inflammatory signs from the underlying destructive process, complicating the interpretation of inflammation-based diagnostic parameters [30,31].

This has important implications for classification, particularly in the interpretation of gingival inflammation and disease activity. Reduced bleeding scores in smokers should not be interpreted as evidence of disease quiescence without consideration of structural indicators. In such contexts, reliance on inflammatory indices alone may lead to underestimation of disease severity and misinterpretation of disease status. Recognizing that smoking alters not only disease risk but also its clinical expression reinforces the need for a more integrated diagnostic approach in which structural and historical parameters are interpreted alongside, rather than subordinate to, observable inflammatory signs.

## 9. Resolving Discordance Between Tissue Behavior and Risk Modifiers

**Figure 2:**
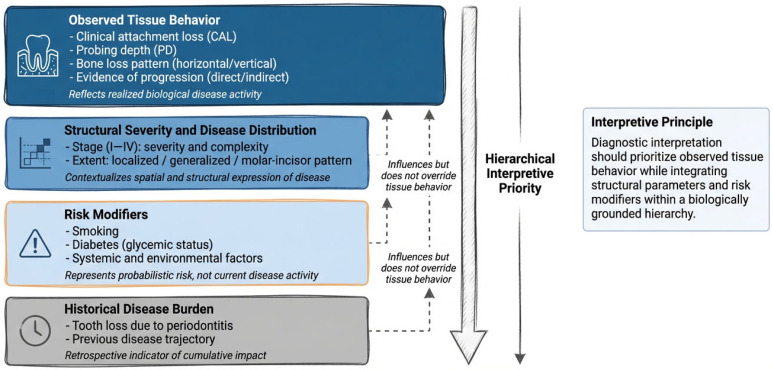
Hierarchical framework for the interpretation of periodontal classification components. The figure illustrates a biologically grounded hierarchical model for resolving interpretive challenges within the 2018 periodontal classification system. Observed tissue behavior, including clinical attachment loss, probing depth, bone loss patterns, and evidence of progression, serves as the primary anchor of diagnosis, reflecting realized disease activity. Structural severity and disease distribution, represented by staging and extent, provide contextual information regarding the spatial and anatomical expression of disease. Risk modifiers, such as smoking, glycemic status, and systemic or environmental factors, represent probabilistic influences on disease susceptibility and potential progression but do not directly reflect current tissue behavior. Historical disease burden, including tooth loss and prior disease trajectory, is incorporated as a retrospective indicator of cumulative impact rather than active disease status. The model emphasizes that diagnostic interpretation should prioritize biological evidence of tissue destruction while integrating structural parameters and modifiers within a coherent hierarchical framework. The downward arrows indicate the hierarchy of interpretive priority, with observed tissue behavior taking precedence during diagnosis, whereas the side annotations emphasize that lower-order factors may influence, but should not override, direct evidence of current tissue destruction. This figure is a conceptual schematic of the interpretive hierarchy rather than a clinical image. Figure created with FigureLab.

In clinical practice, discrepancies may arise when tissue destruction and risk modifiers suggest different grades (Figure 2). For example, rapid bone loss relative to age may indicate a higher grade despite limited exposure to traditional risk factors, whereas significant systemic risk may be present in patients with relatively modest structural destruction. These situations reflect the distinction between observed biological behavior, representing realized disease outcomes, and probabilistic risk, representing the potential for future progression [32].

A hierarchical interpretation provides a coherent resolution. Within this framework, evidence of tissue destruction, whether directly observed or indirectly inferred, serves as the primary anchor of grading [2]. Structural severity and distribution provide contextual information regarding the anatomical and spatial expression of disease, while systemic and behavioral modifiers refine the interpretation by indicating susceptibility and potential trajectory [2]. Such an approach is particularly important in cases where modifier burden and observed destruction are poorly aligned. High-risk exposure without corresponding structural damage may indicate potential rather than realized progression, whereas severe destruction in the apparent absence of major modifiers may reflect unrecognized or historical influences. These scenarios should not be viewed as contradictions but as expressions of the probabilistic relationship between susceptibility and disease manifestation. By prioritizing tissue-based evidence while integrating modifier information in a contextual manner, the classification retains its biological grounding and clinical coherence. This approach is intended to help grading reflect observed disease behavior rather than being disproportionately driven by risk factors alone, and may thereby support more accurate and meaningful clinical interpretation. Prioritizing observed tissue destruction over modifier-based interpretation is advanced here as a proposed interpretive principle rather than an established finding: comparative evidence demonstrating that it improves diagnostic accuracy, prognostic validity, or treatment outcomes does not yet exist and is needed.

## 10. Toward a Phenotype-Oriented Interpretation

The apparent inconsistencies encountered in staging and grading arise when heterogeneous disease presentations are compressed into single categorical labels. Periodontitis encompasses a spectrum of phenotypes influenced by local, systemic, and behavioral factors, and these dimensions are not fully captured by categorical classification alone [33].

A phenotype-oriented approach recognizes that focal and generalized disease patterns may reflect distinct biological processes, that historical outcomes such as tooth loss may not align with current disease activity, and that susceptibility factors and realized tissue destruction represent different dimensions of disease expression. Interpreting these dimensions in isolation risks oversimplification, whereas integrating them within a structured framework allows for a more nuanced understanding of disease behavior.

Importantly, a phenotype-oriented interpretation does not replace the classification system but rather refines its clinical application. Patients with similar stage and grade assignments may differ substantially in the organization, distribution, and activity of disease, as well as in the influence of systemic and behavioral modifiers. Recognizing these differences allows the clinician to move beyond categorical labeling toward a more individualized characterization of disease, which may have implications for prognosis, treatment planning, and maintenance strategies.

In this context, phenotype-oriented interpretation represents an evolution in the use of the classification, aligning it more closely with the biological complexity of periodontitis while preserving its value as a standardized diagnostic framework.

The same interpretive tension can be recognized, by analogy, in peri-implant conditions, although a systematic treatment lies beyond the scope of this Perspective. Peri-implant marginal bone loss is a historical structural endpoint that records cumulative loss of support, much as periodontal attachment loss does, whereas current peri-implant disease activity is defined separately by clinical signs such as bleeding or suppuration on probing, which characterize peri-implant mucositis and peri-implantitis. A structural outcome at an implant therefore need not coincide with current inflammatory activity, paralleling the periodontal distinction between past destruction and present disease status. Systematic application of the proposed hierarchy to peri-implant diagnosis would require dedicated development and validation and is identified here only as a direction for future work.

## 11. Treated Versus Untreated Periodontitis: An Under-Emphasized Clinical Distinction

A further point concerns how the current classification conveys the difference between untreated active disease and previously treated, stabilized periodontitis. Importantly, the 2018 framework, together with subsequent European Federation of Periodontology guidance, does provide a disease-status overlay for this purpose: a treated patient is described, for example, as having “Stage III Grade B periodontitis, currently stable, in remission, or unstable,” with stability defined by the absence of bleeding on probing and shallow residual probing depths. Stage and grade are deliberately retained as lifelong cumulative descriptors precisely because current disease activity is conveyed separately through this status overlay. The limitation is therefore not that the framework lacks this distinction but that the status descriptors are under-used and insufficiently emphasized in routine reporting, education, and research, so that staging is frequently cited in isolation [2].

From a biological perspective, these states are fundamentally different. Untreated periodontitis is characterized by active inflammation, ongoing host–microbial dysregulation, and continued risk of tissue breakdown [15]. In contrast, treated and stabilized periodontitis reflects a controlled inflammatory environment, with reduced microbial burden and maintenance of periodontal stability under supportive care. Despite these differences, both conditions may receive similar stage and grade assignments based on structural and historical criteria [34]. This creates a potential misalignment between classification and current biological status. A patient with previously severe destruction but long-term stability may be categorized similarly to one with active disease progression, despite markedly different clinical needs and prognostic implications. This limitation highlights the importance of complementing structural classification with an assessment of disease activity status.

The absence of this distinction may also influence clinical decision-making and communication. Patients with comparable structural damage may differ significantly in maintenance requirements, risk of recurrence, and therapeutic priorities depending on whether disease is active or controlled. Recognizing this difference would enhance the clinical applicability of the classification and better reflect the dynamic nature of periodontal disease over time.

Expanding on this point clarifies why foregrounding disease status matters clinically. Following active therapy, periodontal stability is maintained through supportive periodontal therapy, and its adequacy is judged using the same status criteria—principally the proportion of sites with bleeding on probing and the persistence of residual probing depths, together with the absence of progressive attachment or bone loss. A patient who retains a high historical stage but presents with minimal bleeding and shallow residual pockets is best described as stable or in remission, whereas comparable structural findings accompanied by widespread bleeding and deep residual pockets indicate unstable disease at materially higher risk of recurrence. This distinction carries direct prognostic and treatment-planning consequences: stable patients can often be maintained at longer recall intervals with monitoring, whereas unstable or recurrence-prone patients require shorter intervals, renewed instrumentation, and reassessment of modifying factors. Recurrence risk is itself influenced by the historical stage, since a previously advanced but now-stable dentition retains reduced periodontal support and therefore less tolerance for renewed breakdown. Because all of this information already exists within the 2018 system, the practical implication is that the disease-status overlay should be reported and acted upon routinely alongside stage and grade, rather than treated as an addition to the framework.

## 12. Practical Application

The proposed hierarchy is intended to operate as a structured second step in interpretation rather than as a replacement for formal classification so that standardization and communication are preserved. In practice, the clinician first records stage, grade, extent, and disease status (stable, in remission, or unstable) exactly as defined by the 2018 system. When the components appear to conflict, the clinician then weights current tissue behavior first, contextualizes it with structural severity and distribution, and uses risk modifiers to refine—rather than redefine—the reading. This sequence changes prognostic and treatment decisions, not the recorded diagnostic label, which avoids introducing a competing nomenclature. For example, two patients both recorded as Stage III Grade B may be managed differently once disease status and current tissue behavior are weighted: a stable patient with no bleeding and shallow residual pockets may be placed on a longer maintenance interval, whereas an unstable patient with persistent bleeding and deep pockets warrants a shorter interval and renewed active therapy—without either patient’s stage or grade being altered.

Two brief scenarios illustrate how the hierarchy resolves common ambiguities. In the first, a patient presents with a single interproximal site showing a deep vertical defect and probing depth consistent with Stage III severity, while the remainder of the dentition is essentially healthy. Recording the extent as localized, and weighting overall current tissue behavior, prevents the single severe site from being read as generalized advanced disease; management is directed at the specific defect rather than at the whole dentition, even though the recorded stage remains III. In the second, a treated patient retains a high historical stage (for example, Stage IV on the basis of past tooth loss) but now presents with a reduced, stable dentition, no bleeding on probing, and shallow residual pockets. Here the disease-status overlay (stable or in remission) and the hierarchy reconcile the apparent “high stage, low current activity” mismatch: the historical stage is retained for the record, while prognosis and maintenance planning are driven by the current stable status. These scenarios are presented as illustrative rather than as data.

## 13. Limitations

Several limitations of the proposed interpretive hierarchy should be acknowledged. First, prioritizing observed tissue behavior increases reliance on clinician judgment and may reduce reproducibility if the instruction to prioritize current tissue behavior is applied inconsistently between examiners. Second, treating risk modifiers as contextual rather than determinative carries a risk of de-emphasizing genuinely informative factors, such as poorly controlled diabetes or heavy smoking, if the hierarchy is applied too rigidly. Third, any interpretive layer introduces potential tension with the standardization that categorical systems are specifically designed to provide. Fourth, and most importantly, no validation data currently exist demonstrating that the proposed hierarchy improves inter-examiner agreement, prognostic accuracy, or treatment outcomes; its value is therefore hypothetical until tested. As noted in the methodology, this work is a conceptual Perspective without original data, quantitative analysis, or systematic synthesis. For these reasons, the hierarchy is intended to complement, and not to override, formal stage and grade assignment, and the present proposals should be read as hypotheses for future empirical evaluation rather than as a validated diagnostic modification.

## 14. Conclusions

The 2018 classification of periodontitis provides a robust and multidimensional framework for diagnosis by integrating severity, complexity, extent, and progression-related considerations. However, its effective clinical application requires recognition that its components reflect different biological dimensions of disease. Apparent discordance most often arises when current tissue destruction, historical outcomes, and risk modifiers are interpreted as equivalent forms of evidence rather than as complementary but distinct indicators.

A biologically grounded interpretive hierarchy offers a coherent resolution by prioritizing observed tissue behavior, contextualizing structural severity and disease distribution, and incorporating risk modifiers without allowing them to override evidence derived from tissue outcomes. Within this framework, classification remains consistent while becoming more aligned with the observed behavior of disease expression.

Beyond resolving diagnostic ambiguities, this approach also highlights the potential for future refinement of periodontal classification. Greater emphasis on phenotype-oriented interpretation, distinction between active and controlled disease states, and integration of biological and clinical variability may enhance the precision and clinical relevance of diagnostic systems. Such developments would extend, rather than replace, the strengths of the current framework, supporting a more nuanced and biologically informed approach to periodontal diagnosis.

## Figures and Tables

**Table 1:** Original components of the 2018 classification (AAP/EFP; Stage I–IV, Grade A–C) and the proposed interpretive reading. The left column states what each component formally encodes; the right column states how the present perspective proposes it be weighted when components conflict.

Component	What the 2018 System Formally Encodes	Proposed Interpretive Reading
Staging (Stage I–IV)	Severity at the worst site (interdental CAL, radiographic bone loss, tooth loss due to periodontitis) plus complexity (probing depth, vertical bone loss, furcation, ridge defects, masticatory dysfunction). Tooth loss ≤4 teeth is a Stage III criterion; ≥5 teeth distinguishes Stage IV.	Read as the peak severity and treatment complexity of the most affected site, interpreted alongside extent rather than as overall disease burden.
Extent/distribution	Localized (<30% of teeth), generalized (≥30%), or molar–incisor pattern.	Treated as an independent distributional phenotype; localized severe disease is biologically distinct from generalized moderate disease and should not be equated with it.
Grading (Grade A–C)	Estimated rate of progression from direct longitudinal evidence or the indirect bone-loss/age ratio (<0.25, Grade A; 0.25–1.0, Grade B; >1.0, Grade C).	Realized progression (direct or indirect tissue evidence) is the primary anchor; grade should reflect expressed disease behavior rather than risk potential.
Grade modifiers	Smoking (cigarettes/day) and glycemic control (HbA1c) shift the grade upward.	Contextual susceptibility factors that refine, but do not redefine, the reading of realized destruction; they indicate the probability of future progression, not proof that it has occurred.
Disease-status overlay	Currently stable/in remission/unstable, defined by bleeding on probing and residual probing depths (EFP guidance).	Conveys current activity separately from the lifelong cumulative stage and grade; should be foregrounded to distinguish controlled from active disease (Section 11).

## Data Availability

No new data were generated or analyzed in support of this research. Data sharing is not applicable to this article.
